# Transcriptomic changes in autophagy-related genes are inversely correlated with inflammation and are associated with multiple sclerosis lesion pathology

**DOI:** 10.1016/j.bbih.2022.100510

**Published:** 2022-09-08

**Authors:** Chairi Misrielal, Astrid M. Alsema, Marion H.C. Wijering, Anneke Miedema, Mario Mauthe, Fulvio Reggiori, Bart J.L. Eggen

**Affiliations:** a Section Molecular Neurobiology, the Netherlands; b Section Molecular Cell Biology, Department of Biomedical Sciences of Cells & Systems, University of Groningen, University Medical Center Groningen, Antonius Deusinglaan 1, 9713AV, Groningen, the Netherlands

**Keywords:** Autophagy, Experimental autoimmune encephalomyelitis, Multiple sclerosis, Neuroinflammation, Mammalian target of rapamycin complex 1

## Abstract

Autophagy is a lysosomal degradative pathway essential for maintaining cellular homeostasis and is also implicated in multiple aspects of both innate and adaptive immunity. Neuroinflammation, along with demyelination and axonal loss, is an important component of multiple sclerosis (MS). Induction of autophagy ameliorated disease progression in experimental autoimmune encephalomyelitis (EAE), a mouse model for MS, underlying a possible link between autophagy and MS pathology. However, it is still unclear how autophagy is affected during different stages of MS. Here, we show a decreased expression of the *autophagy-related* (*ATG*) genes during the acute phase of EAE development in mice as well as in mixed active/inactive lesions of post-mortem human MS brain tissues. Using spatial transcriptomics, we observed that this decreased *ATG* gene expression is most prominent in the core of mixed active/inactive lesions. Furthermore, we observed a hyper-activation of the mammalian target of rapamycin complex 1 (mTORC1) in lesions, which could inhibit both the initiation of autophagy and the transcription factors that regulate the expression of the *ATG* genes. Thus, based on our data, we propose a negative regulation of autophagy in MS, possibly through persistent mTORC1 activation, which depends on the lesion stage. Our results contribute to the understanding of the role of autophagy in different stages of MS pathology and point to the mTORC1 pathway as a potential modulator that likely regulates central nervous system (CNS) homeostasis and neuroinflammation in MS.

## Highlights

• Autophagy is differently regulated in various EAE and MS stages.
• Expression of *ATG* genes inversely correlates with inflammation in EAE mice.
• *ATG* gene expression is decreased in mixed active/inactive brain lesions.
• Targeting mTORC1 may be a promising therapeutic target in MS pathology.

## Graphical abstract

**Figure:**
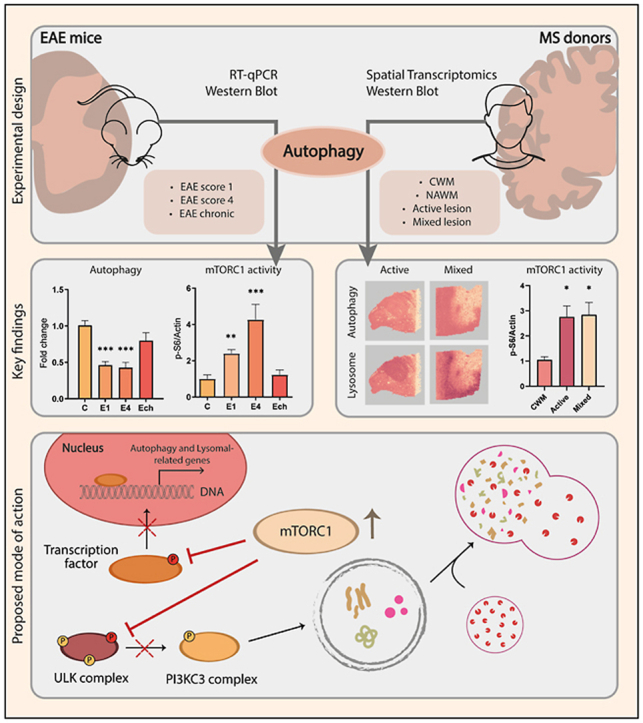

## 1 Introduction

Autophagy is a highly dynamic degradative process that recycles intracellular components, e.g. cytoplasmic proteins, protein complexes, and organelles, via their sequestration within double-membrane vesicles called autophagosomes, and delivers them into the lysosome for turnover (Choi et al. 2013; Lahiri et al. 2019). Autophagosome biogenesis is mediated by the autophagy-related (ATG) proteins and can be subdivided into five steps; (i) initiation and nucleation, (ii) elongation and closure, (iii) maturation, (iv) fusion, and (v) cargo degradation (Misrielal et al., 2020; Nakatogawa 2020). An important regulator of autophagy is the mammalian target of rapamycin complex 1 (mTORC1), a kinase complex that represses autophagy through ULK1 phosphorylation under nutrient-rich conditions (Lahiri et al. 2019; Zachari and Ganley 2017). This represses the activation of the ULK kinase complex, thereby preventing the induction of the downstream ATG machinery. Under starved conditions, in contrast, inhibition of mTORC1 results in the de-repression and induction of autophagy (Lahiri et al. 2019; Zachari and Ganley 2017).

Autophagy is tightly linked to innate and adaptive immunity, in which autophagy contributes to immune responses at multiple levels. Conversely, perturbed autophagy leads to an aggravated response of the immune system (Levine et al. 2011; Yin et al., 2018; Deretic et al. 2013). Besides its function in maintaining cellular homeostasis, there is also a growing interest in understanding the role of autophagy in the context of central nervous system (CNS)-related disorders, since impaired autophagy has been linked to pathological conditions, such as neurodegenerative disorders and autoimmune diseases (Beek et al., , 2018; Yin et al., 2018; Levine and Guido, 2019).

Thus, over the years, research has also addressed the role of autophagy in the pathogenesis of multiple sclerosis (MS), which is driven by environmental, genetic, and immunological factors (Sawcer et al., 2011; Bjornevik et al., 2022; Patergnani et al., 2021). MS is a demyelinating autoimmune disorder of the CNS, which involves innate and adaptive immunity due to the presence of glial and autoreactive T-cells, respectively, and it is characterized by neuroinflammation, demyelination, and progressive axonal loss (Dyment et al., 2006; Sawcer et al., 2011; Dobson and Giovannoni 2019). MS has a range of disease courses, and the majority of MS patients suffer from relapsing-remitting MS, which is characterized by bouts of inflammation and neurodegeneration that ultimately transition into secondary progressive MS (Dobson and Giovannoni 2019). Approximately 10% of MS patients suffer from primary progressive MS, which is characterized by a chronic and progressive worsening of the neurological functions (Miller and SiobhanLeary., 2007). In MS, demyelinated areas, also called lesions, are classified into different types based on the degree of inflammation and demyelination (Van Der Valk and De Groot, 2000; Kuhlmann et al., 2017). This underlines that MS is a heterogeneous disease, in which there is not only variability between patients, but also within one individual. Therefore, it is important to investigate the molecular mechanisms that might contribute to disease activity over the course of MS. Interestingly, a few studies have indicated that autophagy is differently involved in both relapsing and progressive forms of MS since the presence of autophagosomes was only detected in chronic MS patients (Albert et al., 2017). In experimental autoimmune encephalomyelitis (EAE), a widely used experimental mouse model for MS, the auto-immune aspect of MS is mimicked by inducing an immune response against the myelin oligodendrocyte glycoprotein (MOG). Multiple studies have observed a decrease in EAE severity when autophagy was induced through the modulation of mTORC1 activity (Boyao et al., 2019; Feng et al., 2017). As a consequence, autophagy has attracted attention as a potential target for the treatment of MS.

Nonetheless, little is known about how autophagy is affected during MS development and progression. In this study, we addressed this question by taking advantage of the EAE mouse model that captures the autoimmunity, inflammation, and demyelination of axonal tracks, which are key pathological features of MS. To evaluate the autophagic activity during disease development, autophagy progression in the CNS tissue from EAE mice at different time points was measured by analyzing the expression of the autophagy marker proteins, microtubule-associated protein 1 light chain 3 (LC3) and p62/SQSTM1, mTORC1 activity, and transcriptional levels of the ATG genes (Moulis and Vindis 2017). These mouse experiments were complemented with the assessment of autophagy in different lesion stages in human MS brain tissue and identified the location in the lesion in which the expression of *ATG* genes is altered using spatial transcriptomics.

## 2 Methods

### 2.1 Animals

All animal experiments were performed in the Central Animal Facility of the University Medical Center Groningen and approved by the Netherlands Central Committee for Animal Experiments and the University of Groningen. Female C57BL/6 mice were used (Envigo, Harlan, the Netherlands) since the induction and progression of EAE is more consistent in female mice. Mice were group-housed with 5 animals per cage, under a 12/12 h light/dark cycle (lights off at 8 p.m., lights on at 8 a.m.) with ad libitum access to food and water. Mice were housed one week in advance before the start of the experiment to acclimatize to the environment.

### 2.2 EAE induction and evaluation

**Fig. 1:**
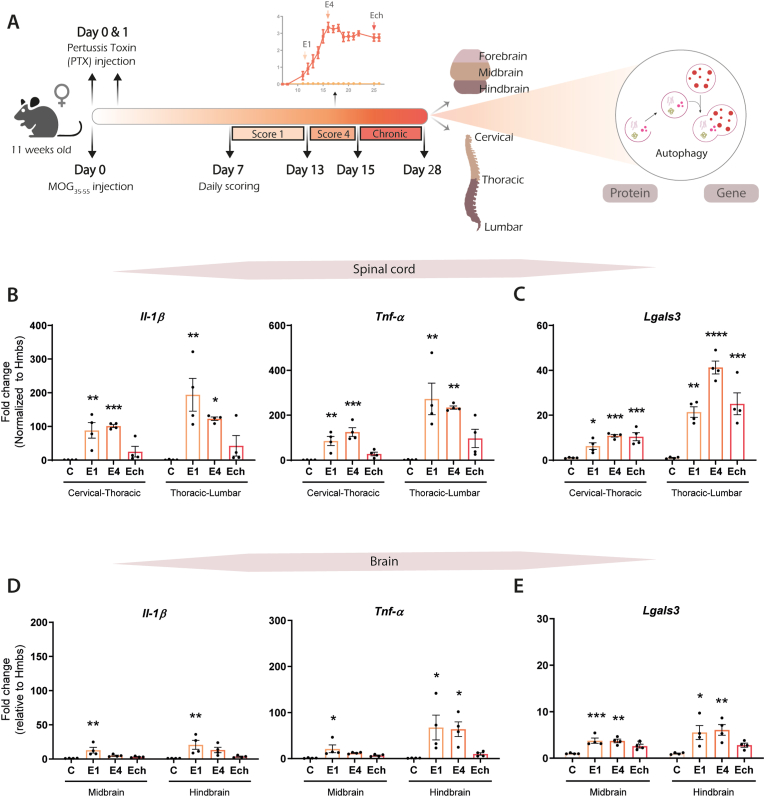
**Inflammatory responses in the spinal cord of EAE mice during the acute phase. (A)** Schematic overview of EAE timeline in which mice were immunized with MOG_35-55_ peptide at day 0 and injected with the pertussis toxin (PTX) at day 0 and day 1. EAE progression was monitored from day 7 until day 28 post injection. Brain and spinal cord tissue were collected from mice that were sacrificed at either score 1 (E1), score 4 (E4), or chronic (Ech), as indicated with arrows, to investigate autophagy at the protein and gene level. Unimmunized mice served as a control (C). **(B-E)** Gene expression levels of Il-1β, Tnf-α, and Lgals3 were determined using qPCR, normalized to Hmbs expression levels, and presented here as fold change compared to control for **(B and C)** spinal cord and **(D and E)** brain tissue. One point represents one mouse (n = 4). Data are presented as mean ± SEM, *p < 0.05; **p < 0.01; ***p < 0.001; ****p < 0.0001 compared to control.

For the EAE experiment, 11 weeks-old female mice were used and immunized with MOG_35-55_ in complete Freund's adjuvant (Hooke, EK-2110) in the upper and lower back, according to the manufacturer's instructions. At the time of immunization, mice were injected intraperitoneal (i.p.) with 150 ng of pertussis toxin (PTX) (Hooke, lot #1008), and this injection was repeated 24 h after the first injection. PTX enhances EAE development by increasing the blood-brain barrier permeability, and the expansion and differentiation of T-cells (Hofstetter et al. 2002). Animals were daily weighted and scored for disease symptoms, using the following scoring scale: 0) no obvious changes, 1) limp tail, 2) limp tail and one-sided mild hind leg weakness, 3) limp tail and moderate hind leg weakness on both sides, 4) limp tail and complete paralysis of hind legs, and 5) moribund. For the experimental study, mice were terminated at score 1 (early symptoms), score 4 (peak of EAE progression), or chronic (3 weeks after onset of symptoms showing slight recovery of EAE development). Unimmunized mice served as the control. Brain and spinal cord tissues were collected, and brain tissue was divided into three regions (i.e., forebrain, midbrain, and hindbrain), while spinal cord tissue was divided into two regions (i.e., cervical and lumbar part) (Fig. 1A). When mice developed their first symptoms, food was put inside the cage. Additionally, as soon as the mice became slightly paralyzed their cage was placed in a 29 ˚C heating cabinet and mice were provided with solid water and powdered food. According to the protocol, mice that lost more than 15% of their body weight were sacrificed and excluded from the study.

### 2.3 cDNA synthesis and quantitative RT-qPCR

Total RNA from mouse tissue sections was isolated using TRIzol (Invitrogen) and mixed with random primers (0.5 μg/μL, Invitrogen). Samples were incubated at 65˚C for 15 min and then kept on ice. Thereafter, a mixture of 8 U/μL of reverse transcriptase (Thermo Scientific), 0.8 U/μL Ribolock RNase inhibitor (Thermo Scientific), 0.5 mM dNTP-mix (Thermo Scientific, R0192), and RevertAid M/MuLV reverse transcriptase buffer (Fermentas) was added to the samples and incubated in a thermal cycler at 42˚C for 1 h, followed by 70˚C for 10 min and the reaction was stopped at 4˚C. Quantitative PCR was performed with a QuantStudio 7 Real-Time PCR system (Thermo Scientific), using the iTaq™ Universal SYBR® Green Supermix kit (Bio-Rad, 1725125). Three technical replicates were included for each sample and hydroxymethylbilane synthase (*Hmbs*) was used as the housekeeping gene. Primer sequences are provided in Table S1. The analysis of the quantitative RT-qPCR was calculated using the 2^−ΔΔCT^ method (Livak and Schmittgen 2001).

### 2.4 Western blot analysis

Brain and spinal cord sections were homogenized in lysis buffer (10 mM Tris, pH 7.4, 2 mM EDTA, 0.25 M sucrose) supplemented with protease (Sigma) and phosphatase inhibitor cocktails (Thermo Scientific). The lysates were incubated on ice for 30 min, vortexed, and centrifuged at 14.000 g for 10 min at 4°C. Protein concentrations were determined using the BCA assay (Thermo Scientific). Equal amounts of protein (i.e., 20 μg) were mixed with Laemmli loading buffer (Bio-Rad) and loaded on a 10% or 18% SDS-PAGE gel. After separation, proteins were transferred onto a polyvinylidene difluoride (PVDF) membrane (Millipore), which were incubated with the following primary antibodies overnight: mouse anti-p62/SQSTM1 (1:1000, Abcam), rabbit anti-LC3B (1:1000, Novus biologicals), mouse anti-S6 (1:500, Cell Signaling), rabbit anti-phospho-S6 (1:500, Cell Signaling), mouse anti- β-tubulin (1:1000, Abcam), and rabbit anti-β-actin (1:1000, Abcam). Thereafter, membranes were washed and incubated with the following secondary antibodies for 1 h at RT: anti-rabbit IRDye800CW and anti-mouse IRDye680RD (LI-COR Biosciences). Signals were detected using the Odyssey Imaging System (LI-COR Biosciences) and densitometric values were determined and quantified at non-saturating exposures using the ImageJ software (Schneider et al. 2012), and normalized against the loading controls.

### 2.5 Spatial transcriptomics

Human post-mortem MS tissues were obtained from the Netherlands Brain Bank and processed with Visium 10x Genomics (catalog no. CG000239 Rev A, 10x Genomics) (*Manuscript in preparation*). Tissue RNA integrity number (RIN) was determined using the Experion RNA STdSens analysis kit applied on the Experion Automated Electrophoresis System (Bio-Rad), according to the manufacturer's instructions. Samples with a RIN value > 6.2 were selected for further processing, the average number of genes per spot, unique molecular identifier (UMI) count, was comparable between samples and independent of the RIN value (Table S2). Thereafter, sectioned frozen brain tissues of 10 μm were mounted on the capture areas of Visium Spatial Gene Expression Slides. Sections were stained for hematoxylin and eosin (H&E) for morphological analysis and spatial alignment of the sequencing data (Fig. S1). Capture areas included spots with a diameter of 55 μm that contained probes with an RNA binding site and a unique spatial barcode resembling tissue location. Tissue sections on the gene expression slide were permeabilized and mRNA was captured on the probes. The generated cDNA, with their specific spatial barcode, were collected as sequencing libraries. The spatial barcodes provided the information to track back the expression in the tissue section for the spatial expression mapping. Four types of white matter tissue sections were included for this study, based on the degree of inflammation and demyelination (Van Der Valk and De Groot, 2000; Kuhlmann et al., 2017; Miedema et al., 2020): control white matter (CWM, n = 2), normal-appearing white matter (NAWM, n = 3), active (n = 4), and mixed active/inactive lesions (n = 5) (Table S2).

Samples were sequenced with an average sequencing depth of 42 million raw reads per sample, using the Illumina NextSeq 500 Sequencing System with NextSeq 500/550 High Output Kit v2.5 (150 cycles). Reads were aligned to the GRCh38 genome and combined with the brightfield images using the default settings in SpaceRanger v1.2.0. Counts were log-normalized with the R package BayesSpace v1.0.0. The H&E staining, together with the myelin basic protein (MBP) expression levels in the tissue samples define the perilesional area (PL) and the core of a lesion (Fig. S1/S2).

**Autophagy and lysosomal gene signature.** The autophagy and lysosomal gene set was chosen from a recent study (Bordi et al., 2021) (Table S2). To highlight the expression of *ATG* genes, the spatial spots were enhanced to a sub spot resolution using the R package BayesSpace v1.0.0. Briefly, each spatial spot is subdivided into 6 sub-spots. Per gene, the expression in sub spots is imputed using an xgboost model that is trained on the expression of the gene of interest in all spots. This results in an imputed log-normalized count estimate per sub-spot. For the *ATG* gene signature and lysosomal signature, the log-normalized count of 20 ATG genes and 25 lysosomal genes was summed per spatial spot. Next, the average *ATG* gene signature in two MS lesion areas (PL and core) was calculated per sample and per area. Data points were plotted using ggplot2 v3.3.5.

### 2.6 Statistical analysis

Statistical analysis was performed using the GraphPad Prism software (v8.4.0) and the one-way ANOVA package followed by Dunnett's multiple comparisons test. Statistical analysis of the spatial transcriptomics data was performed using the paired *t*-test. Data were presented as mean ± standard error of the mean (SEM). The significance level was set to p < 0.05: *p < 0.05, **p < 0.01, ***p < 0.001, ****p < 0.0001.

## 3 Results

### 3.1 EAE mice display inflammatory features predominantly in the spinal cord

EAE is a commonly used model for the inflammatory phase of MS (Constantinescu et al., 2011). In this study, EAE was induced in female mice and the CNS tissue was collected at score 1 (E1), score 4 (E4), and the chronic phase (Ech), to investigate changes in autophagic activity at both the protein and gene expression levels. The mouse CNS was separated into brain (forebrain, midbrain, hindbrain) and spinal cord (cervical and thoracic parts) samples (Fig. 1A). Since EAE has minor effects on the forebrain (Constantinescu et al., 2011), we excluded this region from our analyses.

In both the thoracic and lumbar parts of the spinal cord from EAE mice, mRNA expression levels of inflammatory cytokines *Il-1β* and *Tnf-α* were determined. These two genes have previously been reported to be induced during EAE and are therefore used as markers to monitor EAE stages (Vainchtein et al., 2014). As expected, gene expression of *Il-1β* and *Tnf-α* was significantly increased during the acute EAE phase (E1 and E4) and returned to control levels during the chronic phase (Ech) (Fig. 1B). In contrast, *Lgals3* mRNA expression gene encoding for Galactin3, a marker for activated astrocytes, microglia, and macrophages, was increased in the acute phase and remained significantly elevated during chronic EAE (Fig. 1C). Most of the EAE-associated pathology is localized in the spinal cord, while the brain is less affected (Constantinescu et al., 2011). This is in line with our findings, in which the expression levels of pro-inflammatory cytokines (Fig. 1D) and the activation of glial cells and macrophages (Fig. 1E) were less extensive in the brain of EAE mice compared to the spinal cord. Altogether, these data confirm the inflammation in the CNS tissue of EAE mice, which was predominant in the spinal cord during the acute phase of EAE.

### 3.2 Dysregulated autophagy during the peak of EAE development is only restricted to the spinal cord

Most of the research modulating autophagy has focused on the peak of EAE, which corresponds to E4 in this study. EAE follows a progressive course, in which the acute phase is characterized by a high inflammatory state, demyelination, and axonal loss (Constantinescu et al., 2011; Vainchtein et al., 2014). To address differences in autophagy during different stages of EAE, we determined the steady-state levels of the ATG proteins over the course of EAE in the brain and spinal cord sections.

**Fig. 2:**
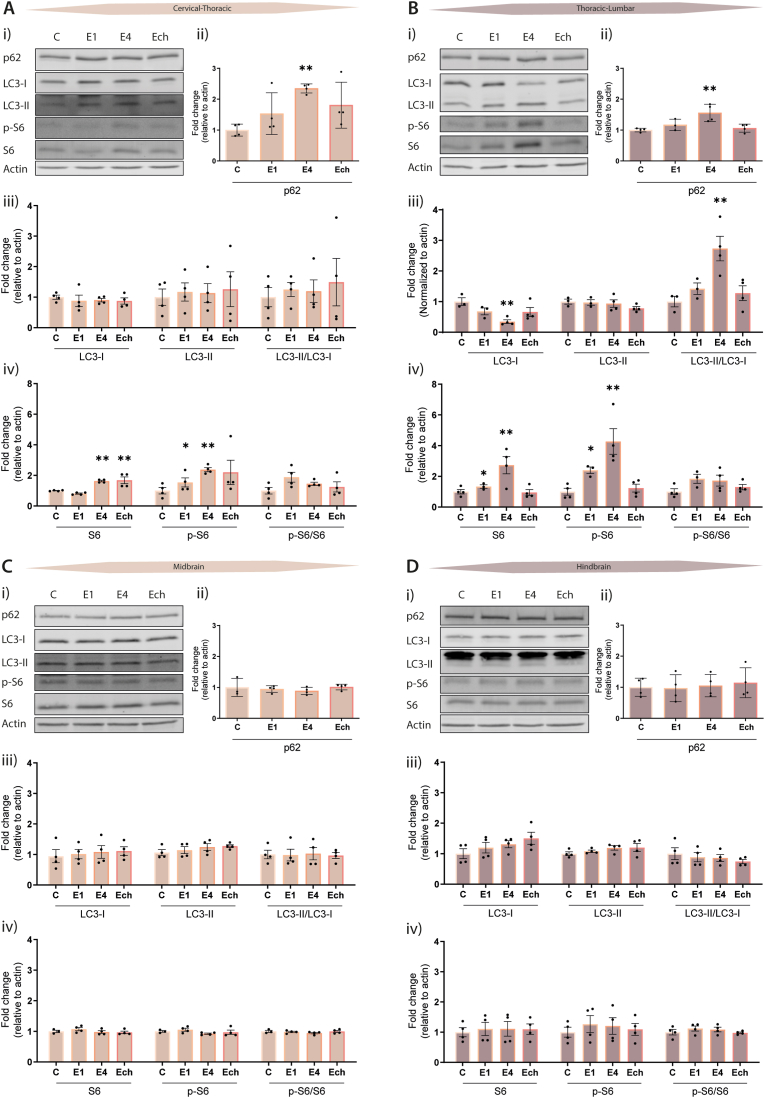
**Alterations of autophagy and increased mTORC1 activity in spinal cords at the peak of EAE development. (A-D) i)** Representative Western blot of LC3, p62, total S6 ribosomal protein, and p-S6 at various stages of EAE in the spinal cord and brain sections; **(A)** cervical-thoracic, **(B)** thoracic-lumbar, **(C)** midbrain, and **(D)** hindbrain. **ii)** Western blot quantification of p62 levels, **iii)** LC3 levels, and **iv)** total S6 ribosomal and p-S6. Levels are normalized to actin and presented as fold change compared to control. One point represents one mouse (n = 3–4). C: control mice, E1: score 1, E4: score 4, and Ech: chronic stage. Data are represented as mean ± SEM, *p < 0.05; **p < 0.01 compared to control.

The analysis of the LC3 proteins provide information about the autophagic state because its conversion from the non-lipidated (LC3-I) to the PE-lipidated form (LC3-II) measures the formation where the overall level of LC3-II measures the number of autophagosomes (Mizushima et al. 2010). p62/SQSTM1, an autophagy receptor (Liu et al., 2016), is another protein that allows analysis of autophagic activity since p62 is degraded by autolysosomes when autophagy is activated and therefore accumulation of p62 correlates with an impaired autophagic flux (Mizushima et al. 2010). Thus, measuring the levels of these proteins provided us with information about autophagy progression in EAE mice (Fig. 2). In both parts of the spinal cord (cervical and lumbar), a significant elevated accumulation of p62 was observed at E4, which returned to control levels at the chronic phase (Fig. 2A_ii_/B_ii_) suggesting either a block in cargo degradation or induction of autophagy at the peak of EAE development (E4). In contrast, LC3 levels were only changed in the lumbar part of the spinal cord, in which the LC3-II/LC3-I ratio was significantly increased at E4 (Fig. 2A_iii_/B_iii_) indicating enhanced autophagosome formation.

To assess whether autophagy is induced or inhibited, we measured the mTORC1 activity during EAE progression, since autophagy is negatively regulated by this complex (He and Klionsky 2009). To determine mTORC1 activity, we measured the phosphorylation status of the S6 ribosomal protein (Chowdhury and Köhler 2015) and observed increased levels of S6 protein and its phosphorylated form (p-S6) in the spinal cord of EAE mice (Fig. 2A_iv_/B_iv_). This increase was observed in EAE mice, which was more prominent in the acute phase of EAE (i.e., E1 and E4). This higher phosphorylation at the peak of EAE indicates an increased mTORC1 activity, which in turn could negatively affect autophagy.

Since we also detected minor inflammation in the brain, we analysed whether autophagy was affected in the mid- and hindbrain of EAE mice. This analysis showed no obvious changes in the p62 and LC3 levels, as well as mTORC1 activity, in these tissues (Fig. 2C/D). Altogether, we found that autophagy is differentially affected in EAE mice. Increased activity in the spinal cord of the negative regulator of autophagy, mTORC1, suggests that autophagy is probably inhibited in the most inflamed regions of EAE mice. Significant changes were seen in the spinal cord at E1 and E4, whereas the levels in this tissue during the chronic phase resembled those of unimmunized control mice. In contrast, the levels in the brain remained unaffected throughout all the EAE phases.

### 3.3 Expression of *ATG* genes inversely correlates with inflammation in the spinal cords of EAE mice

Next, we explored whether the expression of several *ATG* genes, as a measure for autophagy activity (Klionsky et al., 2021; Bordi et al., 2021), was transcriptionally affected during EAE development.

**Fig. 3:**
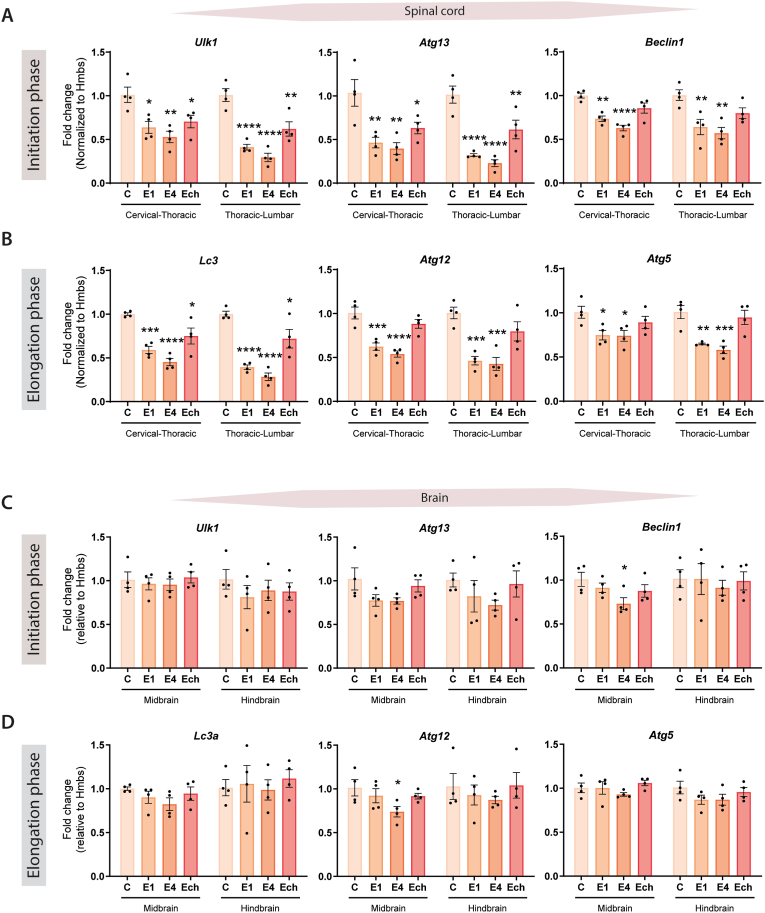
**Decreased expression of ATG genes in the spinal cord of EAE mice at various stages.** Quantitative RT-PCR analysis of genes involved in the **(A and C)** initiation phase of autophagy and **(B and D)** elongation phase for control and various stages of EAE in the spinal cord and brain tissue of mice. mRNA expression levels were normalized to Hmbs and presented as fold change compared to control. One point represents one mouse (n = 4). C: control mice, E1: score 1, E4: score 4, and Ech: chronic stage. Data are presented as mean ± SEM, *p < 0.05; **p < 0.01; ***p < 0.001; ****p < 0.0001 compared to control.

Expression levels of *ATG* genes involved in either the initiation or elongation and closure step of the autophagosome formation were included for a more complete view of the autophagy process, which were determined in the brain and spinal cord tissue with quantitative RT-PCR. Genes involved in the initiation step of autophagy included *Ulk1, Atg13,* and *Beclin1*, which are important for the induction and phagophore nucleation (Nakatogawa 2020; Misrielal et al., 2020), were significantly downregulated in the spinal cord of EAE mice compared to the controls (Fig. 3A). Similarly, downregulation of *Lc3a*, *Atg12*, and *Atg5*, factors involved in the elongation and closure step during autophagosome formation, were observed in EAE mice (Fig. 3B). During the chronic phase, the expression levels of these *ATG* genes were still reduced, albeit less pronounced. When looking at the expression levels of the same *ATG* genes in the brain, these differences were less prominent, and only the decrease in *Beclin1* and *Atg12* expression was significant in the midbrain at E4 (Fig. 3C/D). Overall, these measurements indicate that autophagy is impaired during EAE development. In particular, we show a reduced expression of different *ATG* genes in EAE mice and most notably in the most inflamed regions, i.e., the spinal cord, during the acute phase of EAE (E1 and E4). This underscores a possible connection between autophagy and inflammation.

### 3.4 Decreased expression of autophagy signature genes in MS lesions

**Fig. 4:**
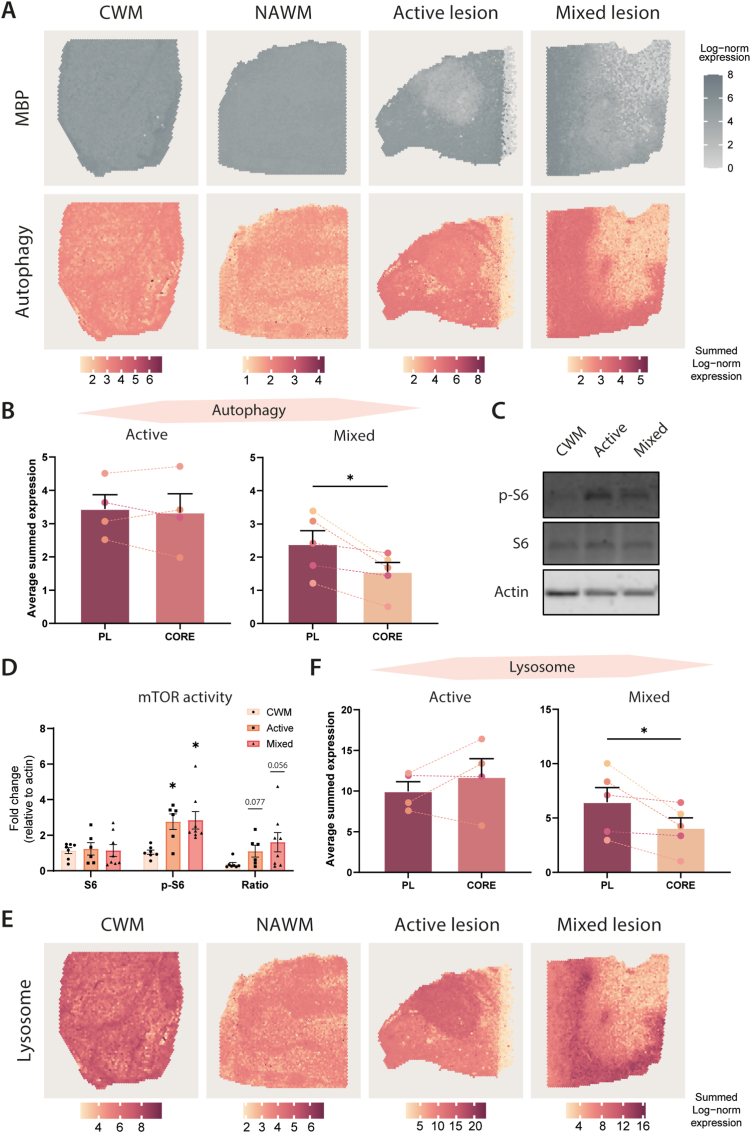
**Decreased expression of ATG genes in the core of mixed active/inactive lesion of human MS donors. (A)** Representative spatial transcriptomics data from human MS brain tissue. MBP expression in CWM, NAWM, active, and mixed active/inactive lesion depicted in grey and shown as log-normalized counts. Expression of the autophagy induction signature is depicted in orange/red and visualized as summed log-normalized counts with their corresponding scale bar underneath. **(B)** Quantification of the autophagy signature in active and mixed active/inactive lesions where the average summed expression is plotted for the areas per lesion sample; perilesional (PL) and core. Data originating from the same sample are connected with a dotted line (n = 3–5 donors) and each color stands for a different donor. **(C)** Representative Western blot of total S6 ribosomal protein and its phosphorylated form p-S6 in CWM, active, and mixed active/inactive lesion samples of MS donors and **(D)** their quantification presented as fold change relative to CWM. Each point represents one donor sample of human MS tissue (n = 5–8). **(E)** Expression of the lysosomal biogenesis signature visualized as summed log-normalized counts with their corresponding scale bar underneath. **(F)** Quantification of the lysosomal signature in active and mixed active/inactive lesions where the average summed expression is plotted per lesion sample for PL and core. Data originating from the same sample are connected with a dotted line (n = 3–5 donors) and each color stands for a different donor. Data are presented as mean ± SEM, *p < 0.05. (For interpretation of the references to color in this figure legend, the reader is referred to the Web version of this article.)

To translate our findings from the EAE model to human MS pathology, we quantified the expression of *ATG* genes in human post-mortem brain tissue sections from MS donors, using the Visium spatial gene expression platform. Moreover, this technique allows to detect regional heterogeneity in MS lesions. White matter lesions were characterized based on immunohistochemical markers of inflammation and demyelination (*Manuscript in preparation*) for which the MBP expression levels in the tissue samples were used to distinguish between the lesion area and surrounding perilesional tissue (Fig. 4A, Fig. S2).

Recently, a set of *ATG* genes has been reported, which can be used to assess whether autophagy is enhanced or inhibited (Bordi et al., 2021). We used this approach to evaluate autophagy progression in lesion types of brain tissue from MS donors. We showed that the summed normalized expression of genes from the autophagy induction list (Table S2) was homogenously distributed throughout the tissue in CWM, NAWM, and active lesions. In the mixed active/inactive lesions, however, clear changes were observed within the lesioned area (Fig. 4A, Fig. S3). To reveal whether differences within the lesion area are significant, we subdivided the active and mixed active/inactive lesion into two regions; the perilesional area (PL), which is the area surrounding the lesion, and the core of the lesion. Quantification of the summed normalized expression of the autophagy induction gene list showed no differences between PL and the core of active lesions, whereas this was significantly decreased in the core of the mixed active/inactive lesion compared to PL (Fig. 4B).

Western blot analysis of CWM, active and mixed active/inactive lesions, showed a higher phosphorylated S6 ribosomal protein level in both lesion types compared to control (Fig. 4C/D). These results indicate a higher mTORC1 activity in lesion areas, which indirectly implies that autophagy is reduced in MS pathology and confirms the spatial transcriptomics data.

To predict whether defects in autophagy are linked to lysosomal dysregulation, we extracted a lysosomal biogenesis signature list (Bordi et al., 2021) and showed the summed normalized expression levels in our human MS Visium dataset (Fig. 4E, Fig. S4). Interestingly, quantification of spatial transcriptomics data of active MS lesions showed an increased expression of the lysosomal signature list in the core of active lesions compared to the perilesional area but it was not significant. In mixed active/inactive lesions, an opposite effect was observed, i.e. a significant decrease in the lysosomal biogenesis signature genes was observed in the core of the lesion (Fig. 4F) similar to the autophagy induction genes.

Altogether, our observations in post-mortem human MS tissues point to a decrease in autophagic activity regulated by mTORC1, which is in line with our observations in EAE mice. In addition, spatial transcriptomics revealed differences in both the autophagy induction and lysosomal biogenesis gene signatures, which depended on the lesion area and were most affected in the core of the lesion.

## 4 Discussion

In this study, we investigated whether autophagy was altered during MS development. We took advantage of the EAE model, an MS mouse model that mostly recapitulates the inflammatory features of MS (Constantinescu et al., 2011). Inflammatory characteristics of the EAE model were observed in our study in which the acute phase is accompanied by the infiltration of peripheral immune cells and extensive activation of glial cells in the CNS, showing a higher inflammatory state in acute compared to chronic EAE (Vainchtein et al., 2014; Borjini et al., 2016; Pyka-Fościak et al., 2020).

In recent years, pharmacological induction of autophagy in EAE mice has been proposed as a potential treatment to reduce the incidence and severity of EAE in mice (Feng et al., 2017; Boyao et al., 2019). However, little is known about the state of autophagy during different EAE progression stages, which is important when this pathway is used as a therapeutic intervention. Our current data showed a disease-dependent decrease in autophagic activity, which is very likely regulated by mTORC1 in both EAE and MS.

We observed a dynamic change in the steady-state expression of ATG proteins, in which the LC3-II/LC3-I ratio and p62 levels are increased in the spinal cord at the peak of EAE. Suppression of LC3-II posttranslational modification and an increase in p62 levels during EAE development have been reported in other studies, but these levels were at the steady-state (Boyao et al. 2019b) and therefore they lack information about the autophagic flux. Lysosomal inhibitors, such as chloroquine, are frequently used to assess the autophagic flux in *in vivo* studies (Klionsky et al., 2021; Moulis and Vindis 2017). However, we did not include this in our *in vivo* experiment since chloroquine also has anti-inflammatory effects (Nirk et al. 2020), which would interfere with our EAE model. This dampening of inflammation by chloroquine could also explain the reduced severity of EAE when mice were treated with chloroquine instead of its effects on autophagy (Bhattacharya et al., 2014). The steady-state levels of the ATG proteins measured in this study are informative when combined with the results about mTORC1 activity. In particular, increased levels of the phosphorylated ribosomal protein S6 during acute EAE indicate an enhancement of mTORC1 activity, which indirectly suggests that autophagy was reduced. This could also explain the observed reduction of LC3-I levels in the lumbar region of the spinal cord at the peak of EAE.

Previous studies investigating autophagy have focused on the levels of ATG proteins (Boyao et al., 2019; Feng et al., 2017). Nowadays, however, measuring *ATG* gene expression levels became easily accessible with techniques like RNA sequencing. Our study highlights the reciprocal link between transcriptional levels of *ATG* genes and the inflammation seen in EAE mice in the spinal cords of the animals during the acute phase of EAE progression. In particular, we observed that autophagy is transcriptionally inhibited during EAE development, something that has not been reported before. However, how *ATG* gene regulation correlates with autophagy induction or activity, remains to be determined.

Whether autophagy is affected by inflammation or vice versa during EAE development is difficult to determine since both can suppress each other through different mechanisms (Deretic 2021). We hypothesize that the decrease in *ATG* gene expression as observed in spinal cord samples, is secondary to EAE-induced inflammation, because we observe that inflammation was also present in the brain while the gene expression of *ATG* genes was much less affected. The observed increase in mTORC1 activity, which is associated with inflammation (Deretic 2021) and their negative regulation of *ATG* gene expression (Chen et al., 2012), corroborates the reported beneficial effects of mTORC1 inhibition in EAE (Boyao et al., 2019; Feng et al., 2017). Interference with mTORC1 activity might be most effective during acute EAE since at this moment mTORC1 activity is highest and the *ATG* gene expression is repressed the most.

To assess whether the altered autophagy as observed in EAE mice also translates to MS in humans, we included different MS lesion stages of post-mortem tissues from MS donors. We took advantage of an autophagy induction signature list, which includes 20 ATG genes that were shown to be upregulated upon autophagy induction (Bordi et al., 2021; Klionsky et al., 2021). Using spatial transcriptomics, we analysed the expression of these *ATG* genes with spatial tissue information. This approach revealed that the expression levels of *ATG* genes were reduced in the core of mixed active/inactive lesions, whereas no change was detected in active lesions. However, the increased phosphorylation levels of the ribosomal S6 protein observed in both lesion types support the argument of increased mTORC1 activity. This data suggests that the increased mTORC1 activity observed in both lesion types precedes the decreased *ATG* gene expression, which is only observed in the core of mixed active/inactive lesions. It will be important to explore whether mTORC1 activity correlates with the expression of *ATG* genes or solely acts via post-transcriptional regulation of autophagy to understand the differences between active and mixed active/inactive lesions. Nevertheless, it is important to note that the decreased expression of *ATG* genes in the core of mixed active/inactive lesions does not directly indicate that autophagy is decreased. It might also be caused by the loss of cells in the demyelinated area that are normally high in autophagic activity as the cellular composition between active and mixed active/inactive lesions is different (Van Der Valk and De Groot, 2000; Kuhlmann et al., 2017; Miedema et al., 2020). Both lesion types show a loss of oligodendrocytes, however, the main difference between the two lesion types is the absence of immune cells in mixed active/inactive lesions. This would explain the decrease in *ATG* gene expression since higher levels of autophagy in peripheral immune cells contributes to cell damage, e.g. by prolonging the survival of autoreactive T-cells (Li et al., 2006; Misrielal et al., 2020).

At last, using a lysosomal biogenesis signature list (Bordi et al., 2021), we observed a decreased expression in the core of mixed active/inactive lesions similar to the *ATG* signature. This suggests a lower lysosomal activity and would also explain the observed accumulation of p62 in EAE mice. Although this does not directly provide information about autophagy, this might indicate that the transcriptional regulation of autophagy and lysosomal function is affected in MS. Two important master regulators of *ATG* and lysosomal genes are the transcription factor EB (TFEB) and E3 (TFE3), which regulate the expression of coordinated lysosomal expression and regulation (CLEAR) motif-containing target genes that are involved in both autophagy and lysosomal biogenesis (Brady et al. 2018). The activity of both TFEB and TFE3 is regulated by mTORC1. Their phosphorylation by mTORC1 results in the association of TFEB and TFE3 with lysosomes, thereby localizing them in the cytosol and inhibiting their activity (Chen et al., 2012; Martina et al., 2014; Brady et al. 2018). This scenario could also very likely explain what we observed, i.e. hyper-activation of mTORC1 inhibits the transcriptional activity of TFEB and/or TFE3 that in turn results in a decreased expression of *ATG* and lysosomal genes.

In conclusion, our data provide novel insights into the regulation of autophagy in EAE and MS, in which we observed that mTORC1 activity is increased and transcriptional expression of *ATG* genes and lysosomal biogenesis genes are decreased, depending on the stage of the disease (i.e. acute EAE and mixed active/inactive MS lesions). We propose that autophagy is transcriptionally inhibited, possibly via the inactivation of transcriptional regulators that regulate the expression of key genes involved in both the induction of autophagy and lysosomal functions. These data contribute to the understanding of how autophagy is affected in different stages of MS pathology, which can be manipulated in future studies to assess the impact on disease severity and development. Altogether, this might identify autophagy modulation as a therapeutic strategy for MS.

## Funding

C. Misrielal is supported by a fellowship from the Graduate School of Medical Sciences of the 10.13039/501100005075University Medical Center Groningen. F. Reggiori is supported by ZonMW TOP (91217002), Open Competition ENW-KLEIN (OCENW.KLEIN.118), and 10.13039/501100001711SNSF Sinergia (CRSII5_189952) grants.

## Declaration of competing interest

The authors declare that they have no known competing financial interests or personal relationships that could have appeared to influence the work reported in this paper.

## Appendix A Supplementary data

Multimedia component 1Primers used for quantitative real-time PCR Multimedia component 1Primers used for quantitative real-time PCR

Multimedia component 2Human MS brain tissue data and autophagy/lysosomal gene set Multimedia component 2Human MS brain tissue data and autophagy/lysosomal gene set

figs1 Hematoxylin-eosin staining in MS tissue. figs1

figs2 MBP expression in MS tissue. figs2

figs3 Expression of *ATG* gene signature in MS tissue. figs3

figs4 Expression of lysosomal gene signature in MS tissue. figs4
