# Supplementary material for: Transcriptomic changes in autophagy-related genes are inversely correlated with inflammation and are associated with multiple sclerosis lesion pathology
Source: Brain Behav Immun Health. 2022 Sep 8;25:100510. doi: 10.1016/j.bbih.2022.100510 (PMC9478930; doi:10.1016/j.bbih.2022.100510)
Supplement: Multimedia component 1Primers used for quantitative real-time PCR [file mmc1.docx]

**Table S1. qPCR primers**

| **Gene name** | **Forward primer 5’-3’** | **Reverse primer 5’-3’** |
| --- | --- | --- |
| **Hmbs** | **CCGAGCCAAGGACCAGGATA** | **CTCCTTCCAGGTGCCTCAGA** |
| **Tnf-a** | **TCTTCTGTCTACTGAACTTCGG** | **AAGATGATCTGAGTGTGAGGG** |
| **Il-1b** | **CCCAAAAGATGAAGGGCTGC** | **TGATACTGCCTGCCTGAAGC** |
| **Lgals3** | **CACTGACGGTGCCCTATGAC** | **AACAATCCTGTTTGCGTTGGG** |
| **Ulk1** | **ACTGTGAAGCAGGTGGTACG** | **TCATCAAGGGCAGCTGATTGT** |
| **Atg13** | **TGGCGGAAGATTTGGACTCC** | **GGGTTTCCACAAAGGCATCG** |
| **Beclin1** | **GAAATCAATGCTGCCTGGGG** | **GGAACAAGTCGGTACCTCTGAA** |
| **Map1lc3a** | **CATCGAGCGCTACAAGGGT** | **GCGGCGCCGGATGAT** |
| **Atg12** | **ACTCTATATGAGTGTTTTGGCAGTG** | **TTCCCACAGCACCGAAATGT** |
| **Atg5** | **CTGCGCCTCTGCAGGACAGT** | **CAAAGCCAAACCGAGGTGCCG** |
