# Supplementary figures and images for: Transcriptomic changes in autophagy-related genes are inversely correlated with inflammation and are associated with multiple sclerosis lesion pathology
Source: Brain Behav Immun Health. 2022 Sep 8;25:100510. doi: 10.1016/j.bbih.2022.100510 (PMC9478930; doi:10.1016/j.bbih.2022.100510)

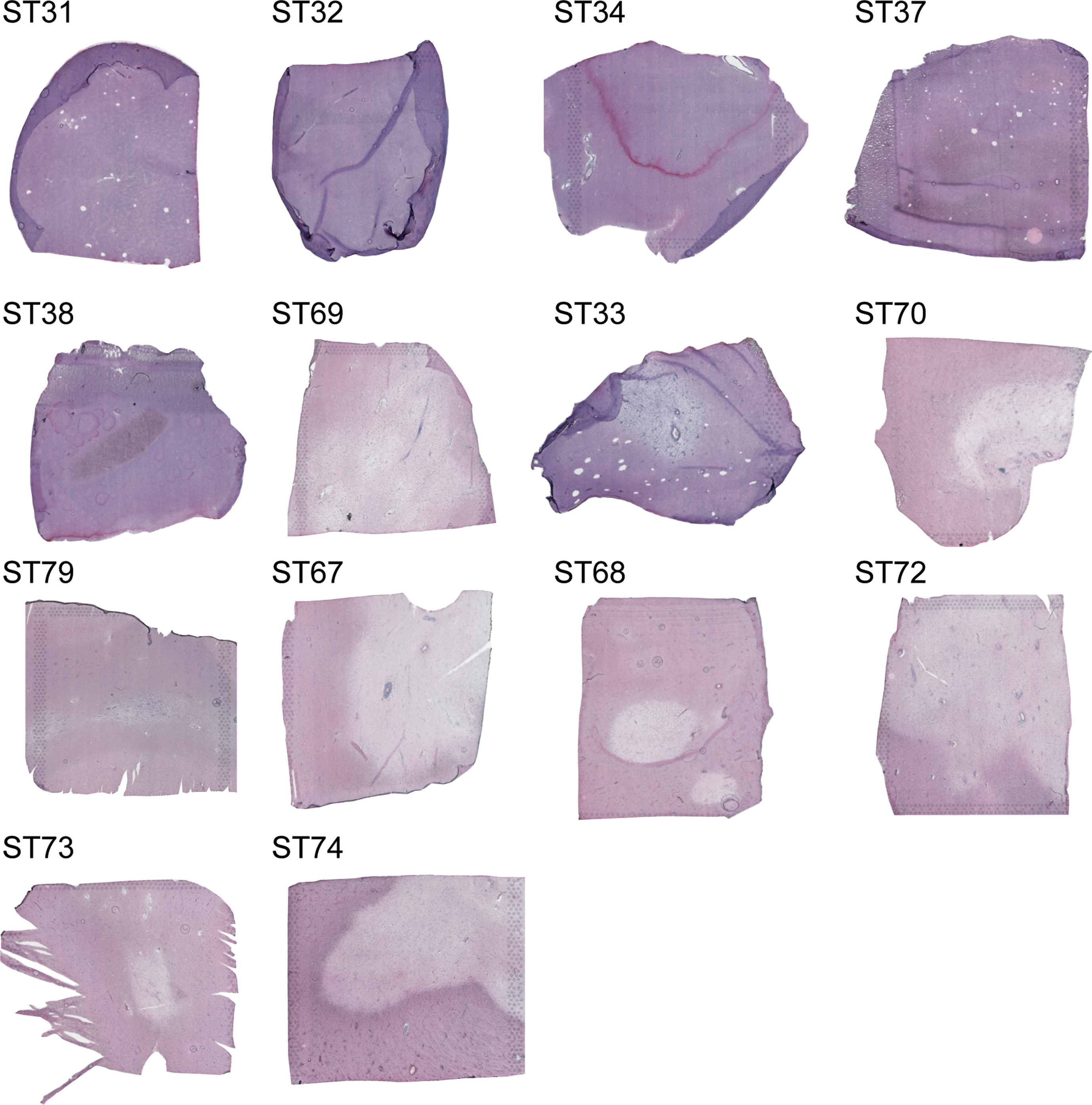

Supplement: figs1 — Hematoxylin-eosin staining in MS tissue. [file mmcfigs1.jpg]

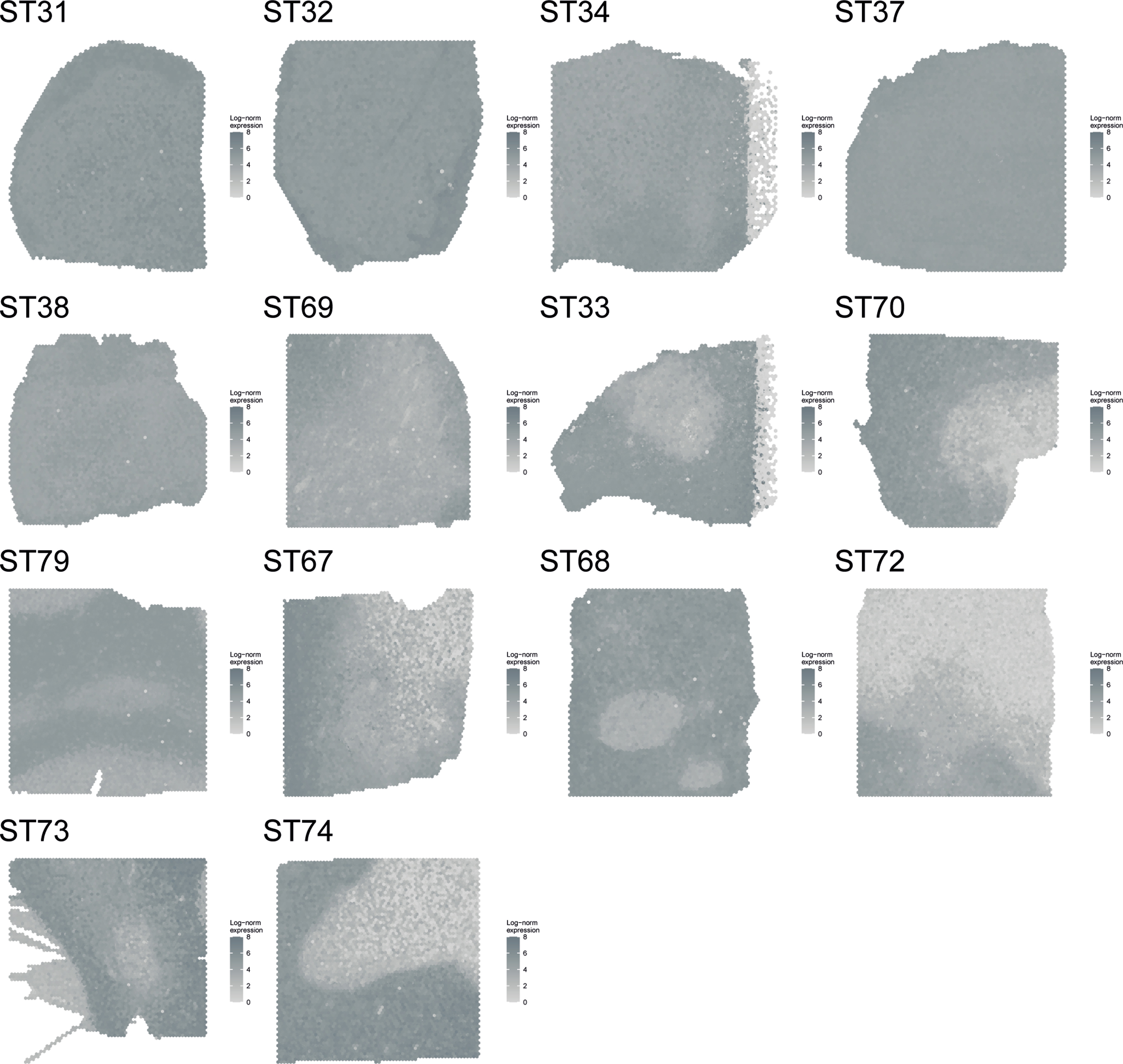

Supplement: figs2 — MBP expression in MS tissue. [file mmcfigs2.jpg]

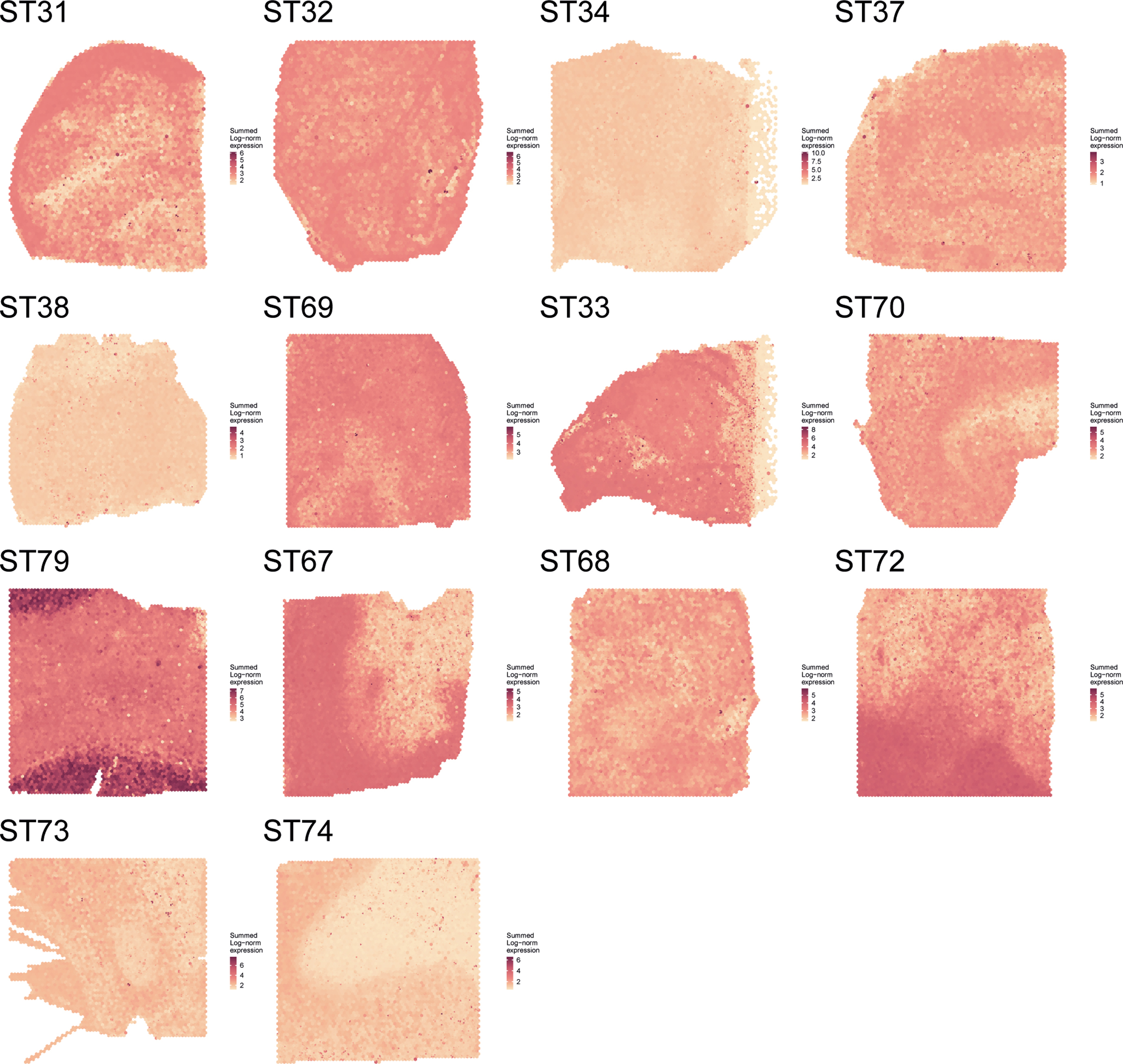

Supplement: figs3 — Expression of ATG gene signature in MS tissue. [file mmcfigs3.jpg]

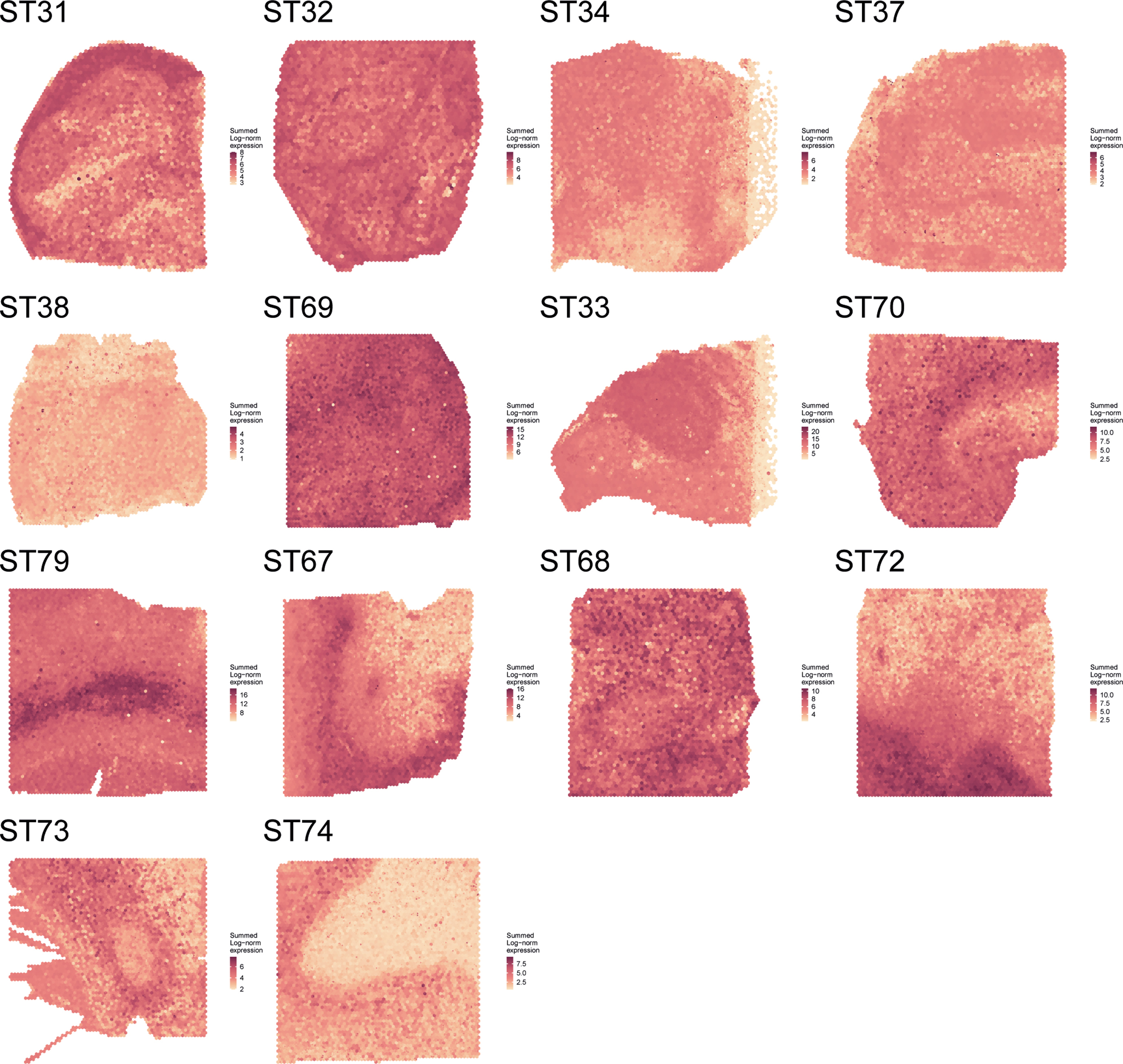

Supplement: figs4 — Expression of lysosomal gene signature in MS tissue. [file mmcfigs4.jpg]
